# Study on the influence mechanism of internet risks on the development risks of Chinese adolescents: the mediating role of digital dependence and the moderating role of digital skills

**DOI:** 10.3389/fpsyg.2025.1699803

**Published:** 2025-11-25

**Authors:** Shan Lv, Yue Hong, Yiyu Huang

**Affiliations:** 1 School of Literature and Media, Dongguan University of Technology, Dongguan, China; 2 Wuhan Municipal Institute of Public Administration, Wuhan, China

**Keywords:** online content risks, online interaction risks, online behavioral risks, development risks, digital dependence, digital skills

## Abstract

**Background:**

For the adolescent group who have grown up alongside the Internet, participating in digital life has become an important part of their daily practices. However, persistent negative online content exposure, interaction experiences and participation in online risky behaviors significant exacerbate adolescents’ developmental risks. This study examined how online content risks, interaction risks, and behavioral risks relate to adolescents’ developmental risks, and whether digital dependence mediates these effects and digital skills moderate them.

**Methods:**

This cross-sectional study was conducted in 2025 among students from five schools in Y and H provinces, China, including one primary school, one junior high school, one senior high school, one junior college, and one university. 899 students (51.4% girls), aged 10–19 (M_age_ = 14.66, standard deviation [SD] = 2.78), participated.

**Results:**

Online content risks, interaction risks, and behavioral risks exert significant positive effects on the digital developmental risks of Chinese adolescents. Furthermore, digital dependence significantly mediated the effects of the three online risks on adolescents’ developmental risks related to alienation from reality. Finally, enhanced digital skills mitigated the positive effect of behavioral risks on digital developmental risks. In addition, the control variables of age, educational level, health status, and economic level were all significant predictors of digital developmental risks.

**Conclusion:**

The results suggest that adolescents’ developmental risks are closely linked to their online experiences, digital dependence, and digital skills. Targeted efforts to reduce harmful online exposure, curb excessive Internet reliance, and strengthen digital competencies may effectively mitigate risks of alienation from reality. Additionally, targeted monitoring and intervention efforts should prioritize those who are older adolescents, have lower educational attainment, are in poorer physical health, or come from economically disadvantaged households.

## 1 Introduction

The proliferation of the Internet and information technology has accelerated the transition into a digital society. For adolescents who have matured alongside the Internet, engagement in digital life constitutes a significant aspect of their daily routines. Data indicate that Chinese adolescents exhibit high frequencies of Internet use, with usage increasing with grade level. For instance, 33.6% of high school/vocational high school students go online daily. Mobile phones are the most commonly used devices among adolescents, although the proportion who own a phone independently remains low. Prevalent Internet use risks include “internet addiction” “materialism and wealth flaunting” and “personal information leakage.” Meanwhile, issues such as “malicious social interaction fraud” “cyberbullying” and “online fraud” though less frequent, are highly detrimental (The People’s Government of Beijing Municipality, 2024). These concerns raise critical questions: What risks do Chinese adolescents face online, and how do these risks influence their personal development?

Adolescence is a period of self-exploration, increasing social independence, and gradual transition into autonomous individuals (Kelly, 2001). In a mediatized society, while digital technology offers considerable convenience and support for adolescents’ daily lives (Bitto Urbanova et al., 2023), limited self-control and judgment during Internet use may expose them to various potential risks, which can escalate into real-life problematic behaviors. Evidence suggests that exposure to harmful content such as violence, pornography, and bullying is detrimental to adolescents’ physical and mental development (Liu et al., 2016; Livingstone and Helsper, 2008). Therefore, systematically identifying the types of online risks adolescents encounter is crucial for understanding their impact.

Online risky behaviors refer to actions that are more likely than other safe online behaviors to result in negative consequences for users (Koutamanis et al., 2015). Previous research has categorized online risks differently. Livingstone and Smith (2014) classified them into content risks (children as recipients of mass-produced content), contact risks (adult-initiated online interactions requiring child participation, sometimes without their knowledge or consent), and behavioral risks (children as participants in peer or networked interactions). Lin (2016) summarized online risks as content risks, contact risks, and commercial risks. These studies indicate that different types of online risks inflict varying degrees of harm on adolescents. However, insufficient attention to risk classification often hinders official data from accurately measuring the online harm adolescents experience. For example, research on child sexual offenses suggests that statistics significantly underestimate potential Internet-related harm (Mitchell et al., 2012). Adolescents’ online behaviors are closely linked to their offline lives; multiple studies indicate that the developmental impact of digital risks stems from the interplay between their real lives and online society (Xu, 2023; Livingstone et al., 2011). Consequently, this study proposes the concept of adolescents’ digital development risks, defined as disruptions to core developmental processes (e.g., identity formation) due to an imbalance between online and offline behavioral influences in digital environments, manifesting as a sense of alienation from reality. Building on the clarification of different online risk types, this study explores their impact on adolescents’ personal development.

Investigating the potential relationship between adolescents’ digital skills and online risks is another key focus. Adolescents’ use of digital skills is diverse and dynamic; they may extensively utilize multiple media functions over time (Zhu et al., 2023). Existing research shows that adolescents do not always easily access and use online services to meet their information needs while avoiding associated risks (Livingstone and Helsper, 2010). Moreover, in today’s digital society, these skills determine how adolescents respond when exposed to digital risks (Smahel et al., 2020). According to the media time displacement theory, media use may negatively affect individual health because time spent on media displaces time allocated to activities conducive to physical and mental development—exemplified by adolescent internet addiction (Kraut et al., 1998). Therefore, examining online risks from the perspective of adolescents’ digital skills is highly significant.

Digital dependence refers to “a person’s continuous and uncontrollable use of digital devices, characterized by a high degree of reliance on them” (Gonçalves et al., 2023). In a digital society, social media, as a key channel for accessing information needed by social systems, has become an integral part of social structures, leading to unprecedented reliance on these platforms. Existing studies indicate that while adolescents are highly dependent on digital technology, they are also aware of its associated risks and often expose themselves to potential dangers (Bitto Urbanova et al., 2023). Previous research has primarily examined specific risk types in isolation, failing to explore the complex relationships between different risks and lacking in-depth investigation into the mediating role of digital dependence.

In summary, our main contributions are threefold. First, based on existing literature, it systematically categorizes three types of online risks—content, interaction, and behavior—and further refines the definition of digital development risks, centering them on reality alienation and core developmental impacts caused by the imbalance between online and real-world behaviors, thereby addressing the conceptual vagueness in prior studies. Second, it overcomes sample selection limitations in previous research by including adolescents from junior high school, senior high school, and university stages, enabling a comprehensive and systematic analysis of the relationships between content risks, interaction risks, behavioral risks, and digital development risks, thus avoiding the narrow focus on a single educational stage or risk type. Third, it employs the Bootstrap model to deeply explore the pathway through which online risks influence digital development risks, while verifying the mediating role of digital dependence and the moderating effect of digital skills, providing more precise empirical support for theoretical mechanism research.

## 2 Theory and hypotheses

### 2.1 Content risks, interaction risks, behavioral risks, and digital development risks

During digital activities, adolescents are highly likely to encounter substantial unfiltered content, with pornographic and violent material being particularly prominent as major content risks. Studies indicate that prolonged exposure to violent and graphic content significantly increases aggressive behaviors among adolescents (Anderson et al., 2017; Zhou, 2020; Shao and Wang, 2019). Adolescence is a critical period for forming self-identity and social cognition; excessive exposure to extremist content can distort their understanding of society, hindering the establishment of correct worldviews and values (Reid Chassiakos et al., 2016). Sustained exposure to such high-risk content can easily lead to socio-emotional developmental disorders, inducing or exacerbating issues such as depression, anxiety (Livingstone and Helsper, 2010; Yang et al., 2023), and low self-esteem (Dhir et al., 2018).

The digital environment has reshaped adolescents’ social patterns, enabling identity construction, social relationship maintenance, and social expression in virtual settings (Livingstone and Blum-Ross, 2017). Online interaction risks primarily include technical risks such as privacy leakage, account hacking, identity theft, and software viruses, as well as virtual social risks like cyberbullying, online sexual grooming, cyber indecency, and online fraud (Livingstone and Helsper, 2010; Lim et al., 2021; Saiphoo et al., 2020; Chou and Edge, 2012). Multiple studies confirm that persistent negative online interaction experiences are significant predictors of mental health and behavioral problems among adolescents (Kowalski et al., 2014; Elhai et al., 2017).

According to social learning theory, adolescents form expectations about behavioral outcomes by observing behaviors (including digital content) and their consequences in their environment, thereby shaping their own cognitions and behaviors (Przepiorka et al., 2019). Behavioral risks such as internet addiction, impulsive consumption, sleep deprivation, and participation in cyberbullying not only directly consume time and energy and affect physical health but also disrupt normal academic, social, and family activities, thereby exacerbating developmental risks (Nesi et al., 2018). Based on this, we propose the following hypothesis.

*Hypothesis 1*: Adolescents’ content risks, interaction risks, and behavioral risks have a significant positive predictive effect on digital development risks.

### 2.2 The mediating role of digital dependence

Digital dependence manifests as excessive and uncontrollable use of smartphones, including high-frequency usage, inability to control usage behavior, excessive focus on phones at the expense of the real environment, psychological withdrawal symptoms such as restlessness, and adverse impacts on interpersonal relationships, learning, and physical and mental health (Liu et al., 2017). Multiple studies have shown that in terms of digital content, addictive digital content, with its strong stimulation and immediate pleasure, easily triggers frequent use and dependence on digital devices among adolescents, squeezing their investment in real-life activities. In interactions, the instant feedback mechanism of virtual socializing can meet adolescents’ psychological needs, leading to over-reliance on online socialization. In terms of digital behavior, increased intensity of online communication among adolescents means weakened offline connections with others, further causing the degradation of their real-world social skills and excessive dependence on digital devices (Valkenburg and Peter, 2011). On this basis, adolescents’ dependence on the digital environment is further intensified, strengthening their mindset of escaping real-world problems through the internet, which may lead to greater digital development risks, such as social isolation. Accordingly, we propose the following hypothesis.

*Hypothesis 2*: Digital dependence plays a mediating role between content risks, interaction risks, behavioral risks, and adolescents’ digital development risks. Specifically, the higher the frequency of adolescents’ exposure to content risks, interaction risks, and behavioral risks, the stronger their digital dependence, and the higher their digital development risks.

### 2.3 The moderating role of digital skills

Not all adolescents exposed to risks suffer harm to the same extent. Digital skills are regarded as key protective factors for adolescents to cope with online risks, which includes operational skills, information search and evaluation capabilities, security protection skills, content creation skills, and critical thinking (Livingstone and Helsper, 2010). Existing studies suggest that digital skills may play a moderating role in digital risk response, which means that they may influence the relationship between risk exposure and negative outcomes. Although individuals with high digital skills tend to have wider online engagement and greater risk exposure, they may face a lower probability of harm (Cabello-Hutt et al., 2018). For instance, a survey of 1,956 students in Hong Kong conducted by Tso et al. showed that digital skills were negatively correlated with risks such as gaming addiction and cyberbullying. Students with high digital skills could more rationally manage device usage time, identify online scams, and reduce bullying risks through technical means (Tso et al., 2022). Digital skills can enhance adolescents’ sense of technical control and confidence in proactive response, making them more inclined to actively address risks rather than passively endure them. This creates the possibility of mitigating the impact of risks. Specifically, adolescents with high-level digital skills can more effectively identify, avoid, and respond to harmful content and interaction risks—such as recognizing misinformation, blocking harassers, setting up privacy protection, and understanding the consequences of online behaviors to reduce their negative impacts. Meanwhile, good operational and content creation skills can also foster positive and constructive online experiences. Although digital skills themselves cannot directly control behaviors, higher-level digital skills may help adolescents make more informed choices, thereby indirectly influencing behavioral patterns. Based on this, we propose the following hypothesis.

*Hypothesis 3*: Digital skills moderate the relationships between content risks, interaction risks, behavioral risks, and adolescents’ development risks.

**Figure 1:**
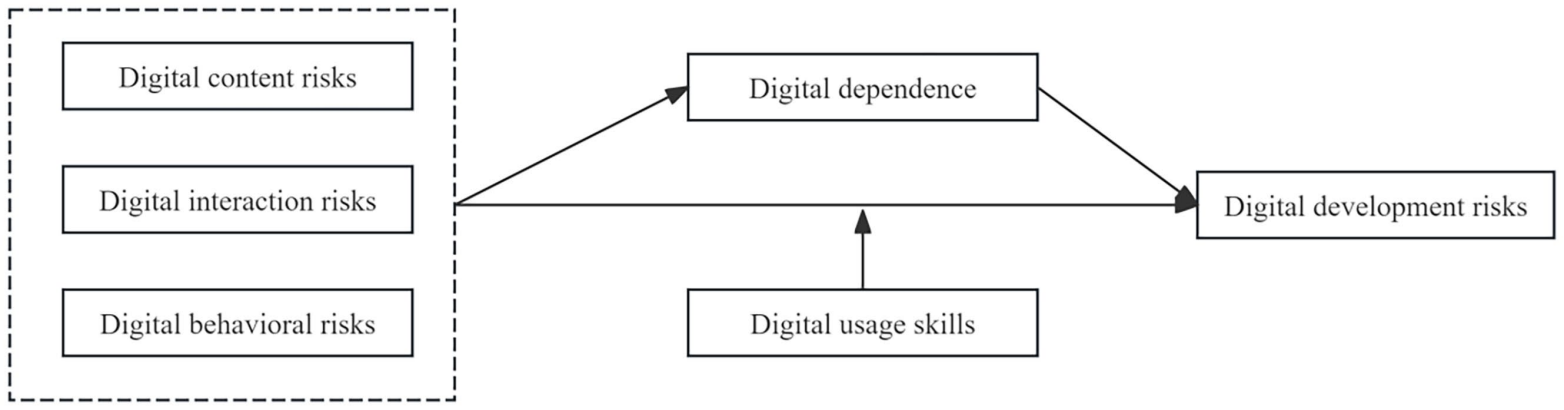
Hypothesis model.

The relationships between variables based on the research hypotheses are shown in Figure 1.

## 3 Methodology

### 3.1 Survey samples

This study collected data using a questionnaire survey. During the pre-survey phase, one university each was selected from Y Province and H Province in China, with questionnaires distributed to first-year undergraduate students via the online platform “Wenjuanxing” (through WeChat groups and Moments). Adhering to the World Health Organization’s definition of adolescents (aged 10–19 years), this study set an upper age limit of 19 years. A total of 183 valid questionnaires were collected from junior college and undergraduate students during this phase.

Subsequently, during the formal survey phase, paper questionnaires were distributed in one primary school and one junior high school in H Province, and one senior high school in Y Province. All returned questionnaires were screened, yielding 215 valid questionnaires from primary school students (H Province), 402 from junior high school students (H Province), and 101 from senior high school students (Y Province) during the formal survey. Merging the valid questionnaires from the pre-survey (183) and formal survey (718), and excluding 2 invalid samples, the final total valid sample size for this study was 899. All participants took part voluntarily and provided written informed consent. The study’s protocol was approved by the Research Ethics Committee of the author’s university.

### 3.2 Variables and measurements

#### 3.2.1 Digital development risks

Existing studies have examined the impact of online risks on adolescent development (Vandoninck et al., 2013; Görzig and Livingstone, 2012) and highlighted the interaction between adolescents’ online behaviors and real life (Livingstone et al., 2011). In Erikson’s life cycle theory, the core developmental task of adolescence is resolving the tension between self-identity and role confusion (Erikson, 1968). Furthermore, developmental actuality theory suggests that healthy adolescent development is achieved through subjective social participation and effective interpersonal interaction, both of which promote social adaptation and reduce the risk of disconnection from reality (Browning, 2011). Studies indicate that adolescents who experience bullying offline are more likely to become targets of cyberbullying (Odgers and Jensen, 2020). Meanwhile, influenced by online media, adolescents’ identities are shaped not only by personal offline experiences but also by subjective online experiences (Xu, 2023). Adolescents with mental health issues in real life tend to browse more negative content and spend more time online rather than engaging in active interaction. Therefore, adolescents’ behavioral performance in real life, which including the quality of interpersonal relationships and online self-disclosure patterns, can serve as strong predictors of their likelihood of encountering online risks (Odgers and Jensen, 2020).

This study measured digital development risks using two items: “Who do you more often share worries or interesting things with?” and “To what extent do you agree that real life is more boring than the online world?” The definition emphasizes that digital development risks, at their core, reflect the potential negative impact on adolescents’ overall development (e.g., identity formation) due to weakened connections with real life and an increased sense of alienation from reality, resulting from the online-offline interaction. Higher total scores (range = 2–10) indicate greater alienation from reality.

#### 3.2.2 Digital content risks

Most scholars subdivide content risks into violent and pornographic content (Kowalski et al., 2014; Zhou, 2020). Livingstone and Smith (2014) defines content risk as digital information that children are passively exposed to and that may cause harm (Livingstone and Smith, 2014). This study assessed content risks using two questions: “How often do you see violent/bloody content (e.g., fighting, self-harm videos)?” and “How often do you see pornographic/vulgar content (e.g., sexually suggestive images)?” Higher total scores indicate greater exposure to content risks.

#### 3.2.3 Digital interaction risks

Livingstone and Smith (2014) defines contact risk as potential harm children face in online interactions with others (including acquaintances or strangers; Livingstone and Smith, 2014). This study measured interaction risks using three questions: “Has your personal information or account been leaked without your consent?” “How often have you been maliciously mocked, insulted, isolated, threatened, or received extortion messages online?” and “Have you received uncomfortable images/videos or been asked to take photos of private parts/perform indecent acts?” Higher total scores indicate greater risks in online interactions.

#### 3.2.4 Digital behavioral risks

Livingstone and Smith (2014) defines conduct risks as online/offline behaviors initiated by children that may harm others or themselves (Livingstone and Smith, 2014). This study measured behavioral risks using two questions: “How often do you insult/attack others in real life?” and “How often do you insult/attack others online?” Higher total scores indicate greater behavioral risks in both online and offline contexts.

#### 3.2.5 Mediating variable: digital dependence

To measure digital dependence, this study drew on scales from previous research, integrating two core dimensions from the Nomophobia Questionnaire (NMP-Q; Yildirim and Correia, 2015), the Social Network Intensity Scale (Ellison et al., 2007), and the Mobile Phone Addiction Scale (Negahban, 2012): self-control over device usage and withdrawal emotional responses. Using a 5-point Likert scale, this study measured two items: “Can you independently control the daily time spent using electronic devices?” and “Would you feel irritable or anxious if you were prohibited from using electronic devices for a long time?” The total score of these two items was used as the indicator of digital dependence.

#### 3.2.6 Moderating variable: digital skills

Digital skills were measured using a multiple-choice question asking respondents whether they could use the following seven skills: finding needed information online, sending instant messages via QQ or WeChat, applying for and using email accounts, downloading and saving various files, setting filters for spam or pop-up ads, removing computer viruses, and self-repairing computer problems. Higher total scores indicate stronger digital skills (Livingstone and Helsper, 2010).

#### 3.2.7 Control variables

Demographic variables included gender, age, whether the respondent is an only child, educational level, self-rated family economic status, and self-rated health status (Livingstone and Helsper, 2010).

### 3.3 Statistical treatment

In this study, the Ordinary Least Squares (OLS) model was used to determine whether there was a significant correlation between digital content risks, digital interaction risks, digital behavioral risks, their interactions with digital usage skills and respondents’ development risks. To study the mediation effects of digital dependence, we used the bootstrap method to test the direct and indirect effects of digital content risks, digital interaction risks and digital behavioral risks, which also reduced potential endogeneity. All statistical analyses were performed with Stata 17.

## 4 Results

### 4.1 Descriptive statistics

The average digital development risks score of the teenagers in the sample was 4.098. Table 1 describes the data and shows that there is a significant correlation between digital content risks, digital interaction risks, digital behavioral risks and respondents’ development risks. In the sample, the frequency of the adolescents’ exposure to content risks, interaction risks, and behavioral risks was generally between “rarely” and “sometimes.” In demographic and social characteristics, 48.5% of the samples were male, 63.3% had siblings, and the average model education level was junior school. The respondents’ self-assessment of economic status was near “average level,” and the self-rated health was close to “good.” On average, the teenagers in the sample possessed more than 3 digital skills.

**Table 1:** Descriptive statistics and correlation coefficient matrix.

Variable	Mean	SD	1	2	3	4	5	6	7	8	9	10	11	12
1. Development risks	4.098	1.546	1											
2. Content risks	1.878	1.636	0.325***	1										
3. Interaction risks	1.229	1.638	0.333***	0.541***	1									
4. Behavioral risks	1.462	1.444	0.325***	0.448***	0.380***	1								
5. Gender	0.486	0.500	−0.00400	0.00300	−0.086**	0.110***	1							
6. Age	14.66	2.779	0.156***	0.382***	0.430***	0.158***	−0.082**	1						
7. Only child	0.633	0.482	0.00900	0.0190	0.0300	−0.0300	−0.164***	0.0380	1					
8. Education	2.280	1.043	0.096***	0.342***	0.409***	0.123***	−0.104***	0.931***	0.0370	1				
9. Economic level	2.190	0.639	−0.087***	−0.147***	−0.152***	−0.103***	−0.060*	−0.0540	0.0170	−0.0350	1			
10. Health condition	2.763	0.830	−0.254***	−0.263***	−0.261***	−0.143***	0.063*	−0.165***	0.0360	−0.161***	0.381***	1		
11. Usage Skills	3.049	1.719	0.186***	0.268***	0.276***	0.191***	0.067**	0.344***	−0.059*	0.321***	−0.062*	−0.085**	1	
12. Digital dependence	3.234	1.944	0.358***	0.496***	0.418***	0.396***	−0.117***	0.397***	0.0210	0.377***	−0.146***	−0.278***	0.189***	1

***p < 0.01, **p < 0.05, *p < 0.1.

### 4.2 Regression analysis

According to the results in Table 2, in Model 1, for each 1-unit increase in content risk, adolescents’ development risk increases by 0.244 units (*p* < 0.01). In Model 2, each 1-unit increase in interaction risk is associated with a 0.274-unit increase in adolescents’ development risk (*p* < 0.01). In Model 3, each 1-unit increase in behavioral risk leads to a 0.288-unit increase in adolescents’ development risk (*p* < 0.01).

**Table 2:** Linear regression results for the influencing factors of digital development risks.

Variable	Development risks					
	Model 1	Model 2	Model 3	Model 4	Model 5	Model 6
Gender	−0.100	−0.030	−0.159	−0.102	−0.032	−0.167*
	(0.100)	(0.099)	(0.099)	(0.100)	(0.099)	(0.099)
Age	0.172***	0.178***	0.184***	0.168***	0.174***	0.185***
	(0.050)	(0.050)	(0.050)	(0.050)	(0.050)	(0.049)
Only child	0.052	0.047	0.082	0.050	0.044	0.069
	(0.100)	(0.100)	(0.099)	(0.100)	(0.100)	(0.099)
Education (Reference group: primary school)						
Middle school	−0.281*	−0.372**	−0.337**	−0.265*	−0.362**	−0.353**
	(0.157)	(0.155)	(0.154)	(0.157)	(0.155)	(0.154)
High school	−0.552*	−0.629**	−0.536*	−0.515	−0.613*	−0.529*
	(0.318)	(0.316)	(0.315)	(0.319)	(0.316)	(0.313)
University and above	−1.559***	−1.740***	−1.452***	−1.520***	−1.724***	−1.487***
	(0.386)	(0.385)	(0.383)	(0.387)	(0.385)	(0.382)
Economic level (Reference group: very poor)						
Poor	−0.274	0.042	−0.381	−0.301	−0.030	−0.417
	(0.466)	(0.466)	(0.462)	(0.466)	(0.468)	(0.460)
General	−0.142	0.183	−0.212	−0.149	0.122	−0.215
	(0.426)	(0.426)	(0.422)	(0.426)	(0.428)	(0.420)
Good	−0.066	0.250	−0.131	−0.071	0.192	−0.108
	(0.437)	(0.437)	(0.433)	(0.437)	(0.439)	(0.431)
Very good	0.154	0.403	0.003	0.144	0.324	0.062
	(0.504)	(0.503)	(0.499)	(0.504)	(0.505)	(0.497)
Health condition (Reference group: very poor)						
Poor	−0.067	0.423	0.037	−0.230	0.146	−0.381
	(0.669)	(0.671)	(0.663)	(0.679)	(0.695)	(0.674)
General	−0.710	−0.223	−0.636	−0.882	−0.494	−1.078*
	(0.630)	(0.635)	(0.625)	(0.643)	(0.659)	(0.639)
Good	−1.145*	−0.675	−1.150*	−1.314**	−0.948	−1.585**
	(0.632)	(0.636)	(0.625)	(0.644)	(0.662)	(0.639)
Very good	−1.282**	−0.810	−1.272**	−1.448**	−1.079	−1.719***
	(0.638)	(0.643)	(0.631)	(0.650)	(0.667)	(0.646)
Digital usage skills	0.094***	0.094***	0.096***	0.138***	0.126***	0.177***
	(0.030)	(0.030)	(0.030)	(0.045)	(0.037)	(0.040)
Content risks	0.244***			0.314***		
	(0.033)			(0.062)		
Interaction risks		0.274***			0.356***	
		(0.034)			(0.065)	
Behavior risks			0.288***			0.467***
			(0.034)			(0.068)
Content risks × Digital usage skills				−0.021		
				(0.016)		
Interaction risks × Digital usage skills					−0.024	
					(0.016)	
Behavior risks × Digital usage skills						−0.052***
						(0.017)
Constant	2.443***	1.756*	2.353***	2.544***	2.041**	2.558***
	(0.903)	(0.906)	(0.895)	(0.905)	(0.925)	(0.893)
Sample size	899	899	899	899	899	899
*R*^2^	0.179	0.188	0.194	0.181	0.190	0.203
Adjusted *R*^2^	0.164	0.174	0.180	0.165	0.175	0.187
*F*	12.05	12.78	13.31	11.45	12.18	13.18

***p < 0.01, **p < 0.05, *p < 0.1.

In Model 1, each 1-unit increase in age is linked to a 0.172-unit increase in adolescents’ development risk (*p* < 0.01), indicating that digital development risk significantly increases with age among adolescents. However, in terms of educational level, with primary school students as the reference group, students in junior high school and above show significantly lower digital development risks (*p* < 0.1; *p* < 0.1; *p* < 0.01). Compared with samples with very poor health status, adolescents who self-rated their health as good or very good have significantly lower digital development risks (*p* < 0.1; *p* < 0.05).

Models 4, 5, and 6 introduce interaction terms between content risk, interaction risk, behavioral risk, and digital skills, respectively. Among these, the main effect of behavioral risk and its interaction effect with digital skills show opposite signs, indicating a significant moderating effect. Specifically, as the frequency of adolescents insulting/attacking others (either in real life or online) increases, their degree of alienation from reality significantly rises; however, improved digital skills can mitigate this negative effect.

**Figure 2:**
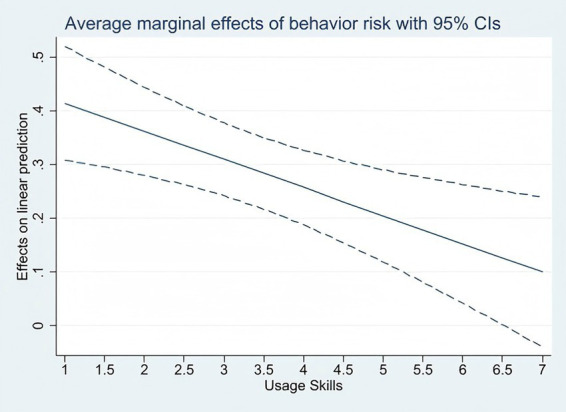
Simple slope diagram.

Figure 2 visually illustrates the interaction effect described above. As adolescents’ digital skills increase, the impact coefficient of behavioral risk on digital development risk decreases. In other words, enhanced digital skills can help adolescents avoid higher levels of digital development risk.

### 4.3 Mediating effect test

This study used the bootstrap method to repeatedly sample 1,000 times to test the mediating effect of content risks, interaction risks and behavioral risks. It can be seen from Table 3 that the confidence intervals for the indirect effects of all did not include 0, and there were intermediary effects.

**Table 3:** Intermediary effect analysis.

Mediator effect	β (95% CI)	SE	*Z*	*p*
Content risks				
Indirect effect	0.088*** (0.060, 0.128)	0.017	5.20	0.000
Direct effect	0.146*** (0.073, 0.219)	0.038	3.83	0.000
Interaction risks				
Indirect effect	0.062*** (0.041, 0.089)	0.012	5.01	0.000
Direct effect	0.198*** (0.124, 0.275)	0.037	5.31	0.000
Behavioral risks				
Indirect effect	0.084*** (0.057, 0.117)	0.015	5.45	0.000
Direct effect	0.201*** (0.116, 0.282)	0.042	4.75	0.000

(1) Control variables in Model 1, Model 2, and Model 3: Gender, Age, Only child, Education, Economic level, Health level, Usage Skills. (2) *p < 0.1, **p < 0.05, ***p < 0.01.

**Figure 3:**
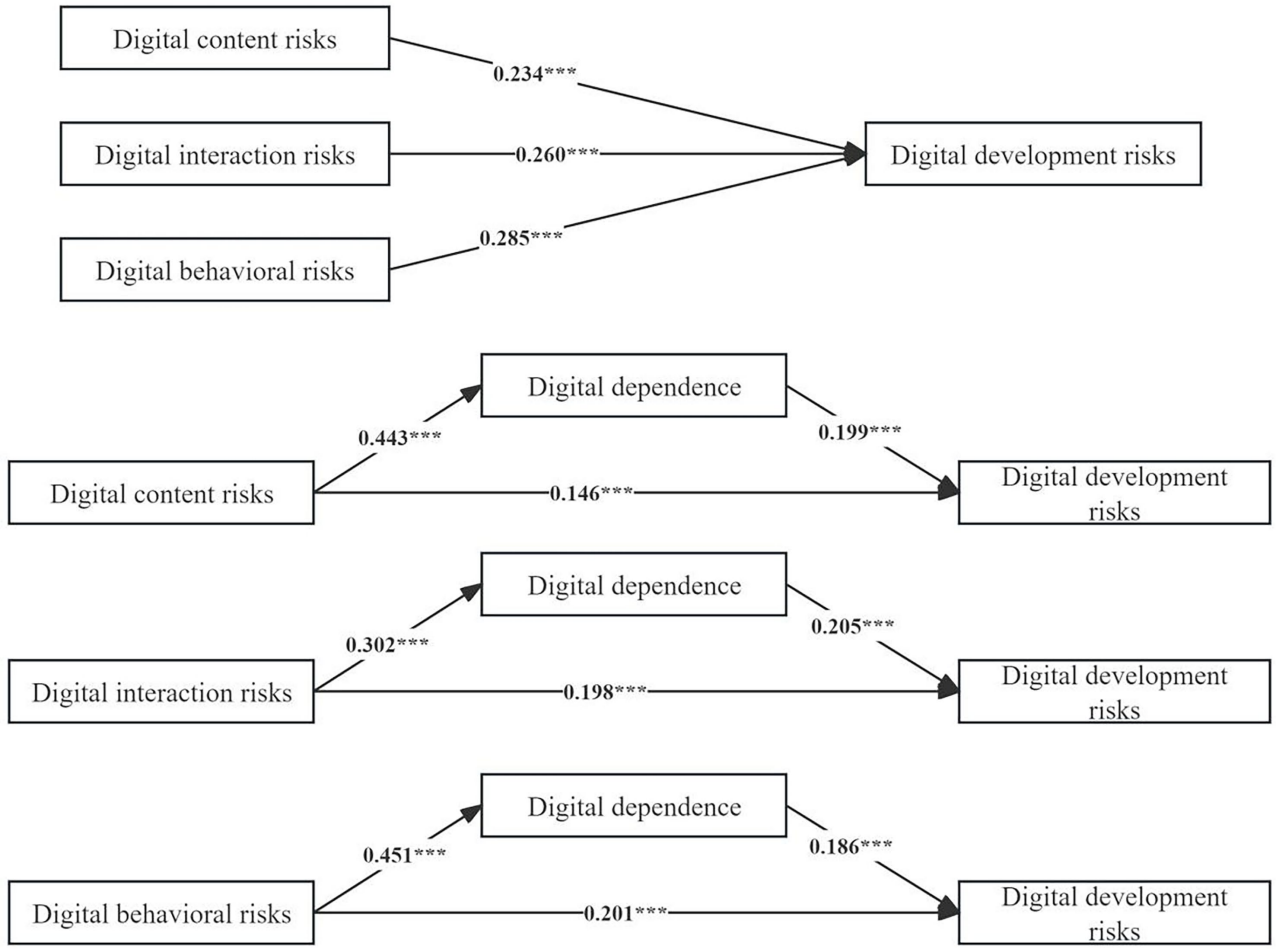
The influence of content risks, interaction risks and behavioral risks on development risks. Different from Table 2, all variables were treated as continuous by default in Table 3 and Figure 3.

Figure 3 illustrates the impact path. When the control variable was included in the model, the positive predictive effect of content risks, interaction risks and behavioral risks on development risks was significant (*β* = 0.234, *p* < 0.001; *β* = 0.260, *p* < 0.001; *β* = 0.285, *p* < 0.001). When digital dependence was included in these models as an intermediary variable, content risks, interaction risks and behavioral risks still had a significant positive predictive effect on development risks (*β* = 0.146, *p* < 0.001; *β* = 0.198, *p* < 0.001; *β* = 0.201, *p* < 0.001). Meanwhile, content risks, interaction risks and behavioral risks had a significant positive effect on digital dependence (*β* = 0.443, *p* < 0.001; *β* = 302, *p* < 0.001; *β* = 0.451, *p* < 0.001), and the latter was associated with higher risk of development risks (*β* = 0.199, *p* < 0.001; *β* = 0.205, *p* < 0.001; *β* = 0.186, *p* < 0.001).

## 5 Discussion

This study aims to examine the relationships between online content risks, interaction risks, behavioral risks, and development risks among Chinese adolescents, and to analyze the mediating and moderating effects involved. In existing literature, research on adolescents’ digital risks has primarily adopted qualitative methods, with few studies systematically exploring different types of digital risks and their relationships using empirical data. Furthermore, this study focuses on the impact pathways of digital development risks and the moderating effect of digital dependence. The results indicate that content risks, interaction risks, and behavioral risks all have a significant positive impact on adolescents’ digital development risks; digital dependence plays a mediating role in these relationships, while digital skills only buffer the negative impact of behavioral risks.

### 5.1 Positive impacts of content risks, interaction risks, and behavioral risks on adolescents’ digital development risks

This study supports Hypothesis 1. It was found that content risks, interaction risks, and behavioral risks all have a significant positive correlation with adolescents’ digital development risks. Specifically, the higher the frequency of adolescents’ exposure to harmful content, the more negative experiences they encounter in online interactions, and the more frequent their engagement in harmful behaviors such as online attacks, the stronger their sense of alienation from the real world. This is consistent with the conclusions of previous scholars (Anderson et al., 2017; Dhir et al., 2018). Among them, behavioral risks have the most prominent impact: actively engaging in aggressive behaviors is more likely to deepen dependence on the virtual environment through social learning effects, thereby exacerbating digital development risks.

A detailed analysis shows that adolescents who are chronically exposed to harmful content such as violence and pornography are more prone to alienation from the real world and tend to be more aggressive. This result supports the findings of existing scholars (Machimbarrena et al., 2018; Zhou, 2020) that adolescents who have experienced cyberbullying are more likely to participate in cyberbullying themselves. Adolescents who have encountered interaction risks such as privacy leakage show significantly weakened real-world social connections, along with higher rates of negative emotional states and mental illnesses, which is consistent with the views of Kowalski et al. (2014) and Elhai et al. (2017). Additionally, adolescents with behavioral risks such as online attacks exhibit a significantly higher degree of dependence on digital devices, further intensifying digital development risks.

### 5.2 Digital dependence plays a significant mediating role

This study supports Hypothesis 2. The results of the mediating effect analysis show that the three types of risks not only directly affect digital development risks but also indirectly increase adolescents’ digital development risks by exacerbating their level of digital dependence. Specifically, addictive information in content risks stimulates adolescents to use digital devices frequently, forming a pathway of “from risk exposure to uncontrolled use, with increased dependence leading to higher development risks.” Some scholars have pointed out that for individuals with social anxiety, introversion, or those who are not good at self-disclosure, the low-pressure environment of online communication can promote self-disclosure, enabling them to establish connections and gain recognition in virtual social interactions (Smith et al., 2021).

The instant feedback in virtual social interactions within interaction risks, although to some extent can compensate for real-world social and socio-emotional development and meet adolescents’ psychological needs (Ellison et al., 2007), excessive reliance on online interactions will still weaken their real-world social skills, leading to alienation from real relationships and triggering greater development risks. The anonymity and immediacy of online behaviors in behavioral risks easily lead to addictive use (Zhou, 2020), and the enhanced sense of dependence further reduces adolescents’ willingness to invest in real life. This mediating pathway clearly reveals the mechanism of digital dependence as a “risk amplifier”—these risk factors, through the reinforcing effect of digital dependence, ultimately exacerbate adolescents’ sense of alienation from reality.

### 5.3 Digital skills play a significant moderating role

This study partially supports Hypothesis 3. The moderating effect of digital skills shows differentiated results. The moderating role of digital skills is significant only in the relationship between behavioral risks and digital development risks: the higher the digital skills, the weaker the negative impact of behavioral risks on digital development risks. This is consistent with the theoretical expectation that digital skills have a protective effect (Vissenberg et al., 2022). Adolescents with high digital skills are more able to recognize the real-world consequences of online behaviors, such as legal risks or social costs of misconduct, and regulate their own behaviors, thereby reducing the damage of behavioral risks to real-world connections.

However, the moderating effects on content risks and digital interaction risks are not significant. This difference may stem from the fact that the impact of content risks relies more on external filtering mechanisms such as platform supervision (Zheng, 2024), while issues in interaction risks such as privacy leakage and cyberbullying are more influenced by the complexity of social environments like peer pressure, making it difficult for individual digital skills alone to fully buffer their negative impacts (Lim et al., 2021).

In addition, as Livingstone and Helsper (2010) noted, “Online skills (and internet literacy conceived more broadly) enables young people to take up new online opportunities and, thereby, encounter more risks.” Digital skills are mainly indirectly related to risks by promoting access to opportunities rather than directly reducing the incidence of risks. This also confirms that the protective effect of skills has boundaries, and its effectiveness is jointly constrained by risk types and external support systems. This emphasizes that the protective effect of digital skills is not universal; relying solely on personally learned and mastered skills is insufficient to cope with various risks on the Internet, and intervention strategies need to be designed in combination with risk types and external systems.

### 5.4 Age, educational level, health status, and economic level as important influencing factors of digital development risks

The impact of control variables is also enlightening. First, we often assume that older adolescents have stronger self-regulation abilities; interestingly, our results show that older adolescents actually face higher digital development risks. We posit that this may be because, as age increases, adolescents expand their scope and frequency of online activities, exposing them to more potential risks. Although their self-regulation abilities may improve, the cumulative effect of risks is more significant, thereby increasing digital development risks. This corroborates the view of Livingstone and Helsper that adolescents improve their online usage behaviors (including access conditions, usage frequency, and online skills) as they grow older. Moreover, adolescents’ self-regulation ability cannot offset risk accumulation—even if this ability improves, it still cannot prevent the increase in online risks.

Second, adolescents with higher educational levels exhibit lower digital development risks. The stronger self-regulation and cognitive judgment abilities they develop through long-term learning help enhance their risk identification and response skills, indicating that improved educational levels can reduce adolescents’ development risks by strengthening cognitive abilities.

Third, adolescents with better self-rated health status have lower digital development risks and weaker sense of alienation from reality. This may be because good physical condition makes them more likely to participate in real-life activities, reducing excessive dependence on the digital environment. This finding supports the research of Bitto Urbanova et al. (2023).

Finally, compared with adolescents who self-rate their family economic level as very poor, those with average or better self-rated family economic levels have significantly lower digital development risks. This may be related to differences in cultural capital accumulation and family education investment (Christensen et al., 2017).

In summary, building on previous studies that classified digital risks into content risks, interaction risks, and behavioral risks (Livingstone and Smith, 2014), this study further proposes the concept of digital development risks, defining them as potential obstacles in adolescents’ emotional, cognitive, personality, and social development (such as alienated identity and sense of alienation from reality) caused by the above three types of risks. It uses quantitative methods to test the impact of these three types of risks on the sense of alienation from reality, a key aspect of digital development risks. Unlike previous studies that mostly focused on the impact of a single risk on individual development, this study constructs a systematic analysis framework covering the relationships among the four types of risks, and introduces the mediating effect of digital dependence and the moderating effect of digital skills. It provides a more comprehensive and systematic exploration of adolescents’ digital risks and their influence mechanisms, offering targeted basis for theoretical construction and practical intervention.

### 5.5 Limitations and future research

This study has certain limitations: First, the samples were only collected from two provinces in China, failing to cover adolescent groups from different regions across the country, which may not fully reflect the overall digital risks faced by Chinese adolescents. Future research could expand the sample coverage to include adolescents from more provinces and regions with varying economic levels to enhance representativeness. Second, as a cross-sectional study, this research cannot draw conclusions about causal relationships. Although relatively stable variables such as age, educational level, health status, and economic level can be regarded as causes in causal relationships, the relationships between content risks, interaction risks, behavioral risks, and digital development risks may be bidirectional. Future studies should adopt experimental or longitudinal research methods to clarify causal relationships. Finally, this study overlooked other potential factors influencing adolescents’ digital development risks, such as family and school environments. Future research could expand the research perspective to analyze the impacts of factors like parental intervention, school-level characteristics, and school restrictions on mobile phone use.

## 6 Conclusion

The main findings of this study are as follows: First, online content risks, interaction risks, and behavioral risks have significant positive impacts on the digital development risks of Chinese adolescents. Second, mediating effect analysis shows that increased exposure to content risks, interaction risks, and behavioral risks strengthens adolescents’ dependence on digital devices and the Internet, thereby increasing their development risks of alienation from reality. Finally, improved digital skills can buffer the positive impact of behavioral risks on digital development risks. Additionally, targeted monitoring and intervention efforts should prioritize those who are older adolescents, have lower educational attainment, are in poorer physical health, or come from economically disadvantaged households.

Based on the empirical findings, we argue that a multi-dimensional collaborative governance framework needs to be established for intervention to systematically prevent and control the risks of adolescents’ mobile Internet use.

First, strengthen the construction of policy regulation and supervision systems. The government should further improve the policy and regulatory system for mobile Internet space governance, focus on implementing special systems for the care and protection of minors, clarify the responsibilities of supervision entities, standards for defining risky content, and norms for intervention procedures. It should also build a full-chain risk prevention and control mechanism to reduce the probability of adolescents being exposed to harmful information such as violence and pornography at the source.

Second, establish a school-society collaborative mechanism for psychological support and behavioral supervision. Schools and society should set up a monitoring system for adolescents’ online behaviors and an assessment system for their mental health, focusing on the frequency and impact scope of typical risk incidents such as cyberbullying, online sexual harassment, and personal information leakage. Risk prevention and control should be strengthened through carriers such as special education courses, psychological intervention hotlines, and case early warning mechanisms. Simultaneously, a home-school linked management system for mobile terminal use should be established to clarify usage scenarios, duration, and content review standards.

Third, enhance the fundamental supporting role of families in risk intervention. Parents should build a regular parent–child communication mechanism and guide minors to transform single-dimensional online social behaviors into diversified real-life participation activities based on their needs, thereby enhancing their sense of connection with real society. Meanwhile, parents need to improve minors’ media literacy through parent–child co-learning and other methods, including information identification ability, privacy protection awareness, and rational usage habits. Empirical data show that adolescents lacking family guidance are more likely to develop online social dependence; their real-life communication skills, social initiative, and environmental adaptability will gradually weaken, eventually leading to an increased sense of alienation from real society.

## Data Availability

The raw data supporting the conclusions of this article will be made available by the authors, without undue reservation.
